# 161. Prospective Study of Cytomegalovirus (CMV) Reactivation in Patients with Multiple Myeloma Receiving anti-CD38 and BCMA Therapies: An Interim Data Analysis

**DOI:** 10.1093/ofid/ofae631.047

**Published:** 2025-01-29

**Authors:** Emily Baneman, Erin Moshier, Larysa Sanchez, Meenakshi M Rana, Samantha E Jacobs

**Affiliations:** Icahn School of Medicine at Mount Sinai, New York, NY; TCI Biostatistics Shared Resource Facility, Institute for Healthcare Delivery Science, Department of Population Health Science & Policy, Icahn School of Medicine at Mount Sinai, New York, NY; Icahn School of Medicine at Mount Sinai, New York, NY; Icahn School of Medicine at Mount Sinai, New York, NY; Icahn School of Medicine at Mount Sinai, New York, NY

## Abstract

**Background:**

The landscape of multiple myeloma (MM) treatment has shifted over the past decade, leading to increased cumulative immunosuppression as patients live longer and receive treatment for multiple relapses. Newer MM therapies, including anti-CD38 and BCMA directed agents, impact cell-mediated immunity. We hypothesized that patients receiving such therapies are at risk of CMV infection and we launched a prospective pilot study to assess CMV incidence in this population.

**Table 1:**
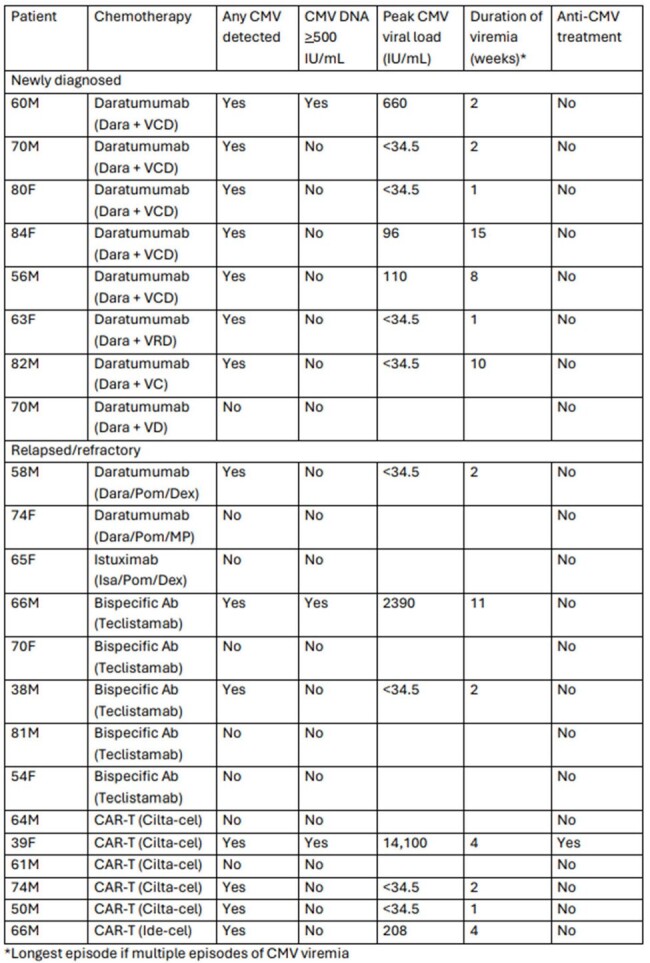
Characteristics of CMV reactivation among patients with multiple myeloma

**Methods:**

Eligible patients included seropositive adults with MM undergoing treatment with anti-CD38 or anti-BCMA therapies. Plasma CMV PCR was monitored every 14 days (+/- 7 days) for three months (Cobas 6800 System, limit of quantification 34.5 IU/mL). The primary endpoint was CMV viral load >500 IU/mL. Treating providers were blinded to results unless CMV PCR was sent outside of the study for clinical reasons.

**Figure 1:**
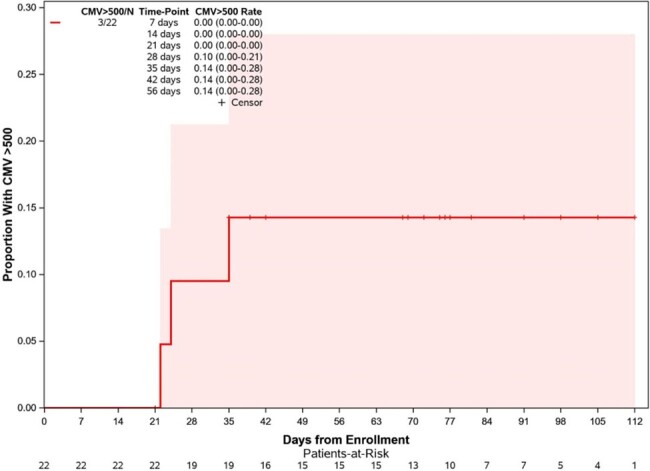
Proportion with CMV > 500 IU/mL during follow-up period

**Results:**

Twenty-two patients had data available at the time of interim analysis. Eleven (50%) patients received anti-CD38 containing regimens (10 daratumumab, 1 isatuximab), 5 (23%) received anti-BCMA bispecific antibody, and 6 (27%) received BCMA-targeted CAR-T. Eight (36%) patients had newly diagnosed MM and 14 (64%) had relapsed disease. Among relapsed patients, median prior lines of chemotherapy was 4 (range 1-13). Nine (41%) patients had prior autologous stem cell transplant. Fourteen (64%) patients had detectable CMV DNA, but CMV viral load remained < 34.5 IU/mL in the majority (8/14, 57%). Only three (13.6%) patients had CMV viral load >500 IU/mL, including one asymptomatic CAR-T cell recipient who received valganciclovir. Median duration of DNA-emia was 2 weeks (range 1-15). There was a trend toward more DNA-emia in subjects with newly diagnosed compared to relapsed MM (88% vs 29%, HR 2.58 [95% CI 0.86-7.79], p=0.0758). Previous history of CMV infection was also associated with CMV reactivation during the study (HR 5.54 [95% CI 1.36 - 22.48], p=0.0063). There were no patients with end-organ CMV disease and no mortality during the study.

**Figure 2:**
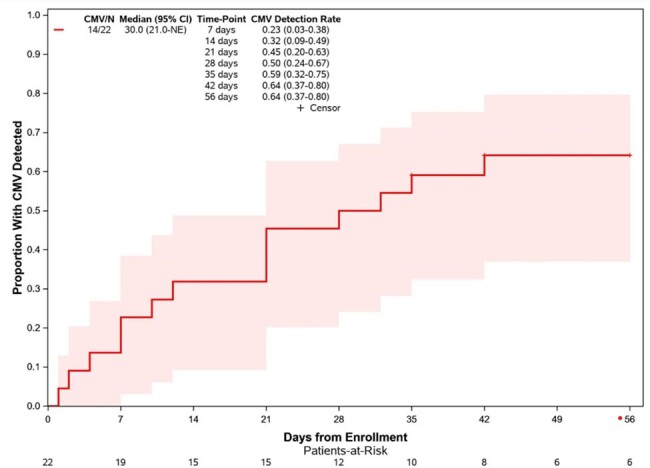
Proportion with any CMV DNA detection during follow-up period

**Conclusion:**

Low-level CMV reactivation occurs frequently in patients with MM receiving anti-CD38 and BCMA therapies, but clinically significant CMV infection remains uncommon. Future studies should also evaluate the impact of subclinical CMV DNA-emia on oncologic outcomes.

**Figure 3:**
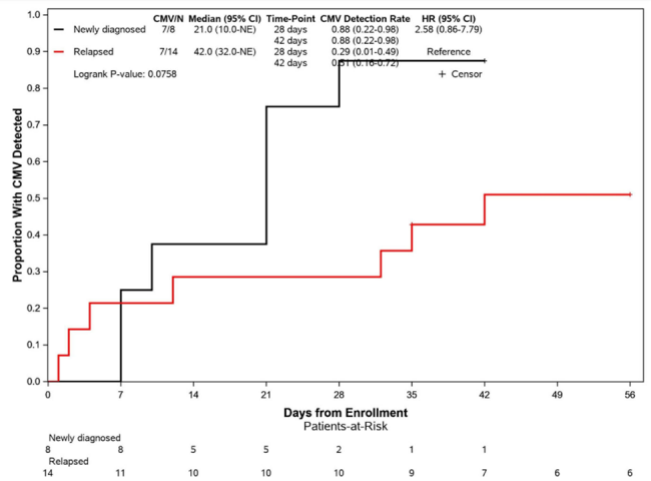
Proportion with any CMV DNA detection, stratified by prior MM treatment

**Disclosures:**

**Emily Baneman, MD**, Merck: Grant/Research Support **Samantha E. Jacobs, MD, MS**, Ansun Biopharma: Advisor/Consultant|Eurofins, Viracor, LLC.: Grant/Research Support

